# Comparing Targeted vs. Untargeted MS^2^ Data-Dependent Acquisition for Peak Annotation in LC–MS Metabolomics

**DOI:** 10.3390/metabo10040126

**Published:** 2020-03-26

**Authors:** Isabel Ten-Doménech, Teresa Martínez-Sena, Marta Moreno-Torres, Juan Daniel Sanjuan-Herráez, José V. Castell, Anna Parra-Llorca, Máximo Vento, Guillermo Quintás, Julia Kuligowski

**Affiliations:** 1 Neonatal Research Unit, Health Research Institute La Fe, 46026 Valencia, Spain; isabel_ten@iislafe.es (I.T.-D.); julia.kuligowski@uv.es (J.K.); 2 Hepatología Experimental, Health Research Institute La Fe, 46026 Valencia, Spain; teresa_martinez@iislafe.es (T.M.-S.); marta_moreno@iislafe.es (M.M.-T.); jose.castell@uv.es (J.V.C.); 3 Health and Biomedicine, Leitat Technological Center, 08028 Barcelona, Spain; jsanjuan@leitat.org; 4 Centro de Investigación Biomédica en Red de Enfermedades Hepáticas y Digestivas (CIBERehd), Instituto de Salud Carlos III, 28029 Madrid, Spain; 5 Departamento de Bioquímica y Biología Molecular, Universidad de Valencia, 46100 Burjassot, Spain; 6 Division of Neonatology, University & Polytechnic Hospital La Fe, 46026 Valencia, Spain; annaparrallorca@gmail.com (A.P.-L.); maximo.vento@uv.es (M.V.); 7 Unidad Analítica, Health Research Institute La Fe, 46026 Valencia, Spain

**Keywords:** liquid chromatography–mass spectrometry, peak annotation, data dependent acquisition, human milk

## Abstract

One of the most widely used strategies for metabolite annotation in untargeted LCMS is based on the analysis of MS^n^ spectra acquired using data-dependent acquisition (DDA), where precursor ions are sequentially selected from MS scans based on user-selected criteria. However, the number of MS^n^ spectra that can be acquired during a chromatogram is limited and a trade-off between analytical speed, sensitivity and coverage must be ensured. In this research, we compare four different strategies for automated MS^2^ DDA, which can be easily implemented in the frame of standard QA/QC workflows for untargeted LC–MS. These strategies consist of (i) DDA in the MS working range; (ii) iterated DDA split into several *m/z* intervals; (iii) dynamic iterated DDA of (pre)selected potentially informative features; and (iv) dynamic iterated DDA of (pre)annotated metabolic features using a reference database. Their performance was assessed using the analysis of human milk samples as model example by comparing the percentage of LC–MS features selected as the precursor ion for MS^2^, the number, and class of annotated features, the speed and confidence of feature annotation, and the number of LC runs required.

## 1. Introduction

Metabolomics is a rapidly evolving field in biomedical research that targets the analysis of the low molecular weight metabolites within a biological system. Hyphenated high resolution liquid chromatography mass spectrometry (LC–MS) is among the most sensitive and selective techniques for the simultaneous analysis of metabolites comprising a wide range of physicochemical properties and concentrations. However, the analysis of untargeted LC–MS data requires the identification or annotation of the metabolites prior to further analysis such as pathway, metabolite enrichment or overrepresentation analysis [1]. An accurate metabolite annotation is key to transform spectral information, first into structural and then into meaningful and consistent biochemical information. The putative identification of a metabolic feature for which the assignment of its structure is highly likely, but not validated through chemical-reference standards, is defined as ‘annotation’ [2]. As the accessibility and analysis of the complete set of potential metabolites is not always feasible, annotation based on MS and MS^n^ information is widely used as a suboptimal alternative. In practice, the comparison of experimentally acquired MS data of a given metabolic feature against a spectral database such as the HMDB (www.hmdb.ca), METLIN (metlin.scripps.edu) or the Kyoto Encyclopedia of Genes and Genomes (KEGG, www.genome.jp) can be used for metabolite annotation. However, MS-based approaches typically lead to multiple molecular formulae for each feature and hence, multiple hits in spectral databases may be obtained. Therefore, standard annotation approaches typically exploit MS^n^ information to refine the number of matches. Nonetheless, the amount of MS^n^ spectra that can be acquired during a chromatogram is limited and a trade-off between analytical speed, sensitivity and coverage must be ensured. One of the most widely used strategies for the acquisition of MS^2^ spectra is data dependent acquisition (DDA), where precursor ions are sequentially selected from full scans based on user-selected criteria such as intensity or charge state during the injection of representative samples, typically at the beginning or end of the analytical batch [1]. The quality and number of acquired MS^2^ spectra depends on additional instrumental parameters such as the spectral acquisition rate, number of precursor candidates selected for fragmentation in each survey MS scan, collision energy, m/z tolerance, precursor widths, and the exclusion time to skip already fragmented ions. A frequently used strategy for metabolite annotation is based on the re-analysis of samples for targeted DDA using a list of precursor ions and retention times selected from the statistical analysis of the data. This strategy limits the reuse of data sets as it requires access to the samples and additional technical bias might also be introduced during sample re-analysis in a separate experiment. So, different DDA MS^2^ experiments have been proposed to increase the coverage of metabolites for which MS^2^ data is acquired. The identification of artefactual features from background contamination and isotopes has been used to generate a preferred ion list to guide precursor selection, thus increasing its efficiency and the MS^2^ coverage [2,3]. Furthermore, the use of time-staggered precursor ion lists for DDA has been proposed to improve the MS^2^ coverage of metabolomes [4]. The use of the integrated application of both collision-induced dissociation (CID) and higher-energy collisional dissociation ion activation methods, multiple different activation energies and narrow precursor ion m/z ranges of 100 or 300 for acquisition of MS^2^ spectra has also been used to provide complementary information and increase the number of unique metabolites for which MS^2^ data is acquired [3].

In this work, we analyze the applicability of two untargeted and two targeted, automated MS^2^ DDA spectra acquisition in the frame of standard quality control/assurance (QC/QA) workflows for untargeted LC–MS metabolomics. These strategies include: (i) untargeted DDA in the MS working range; (ii) untargeted iterated-DDA split into several m/z intervals; (iii) targeted dynamic iterated DDA using an inclusion list of potentially informative LC–MS features; and (iv) targeted dynamic iterated DDA of (pre)annotated features. For (iii) and (iv), LC–MS features were extracted from the injection of two blanks and three QCs acquired during the initial system conditioning included in standard QA/QC protocols. The different DDA acquisition approaches were applied to the annotation of LC–MS data obtained during the analysis of human milk (HM) samples and their performance was assessed by comparing the percentage of LC–MS features selected as precursor ions for MS^2^, the number and class of annotated features, and the number of LC runs required.

## 2. Results and Discussion

### 2.1. Data Overview

**Figure 1:**
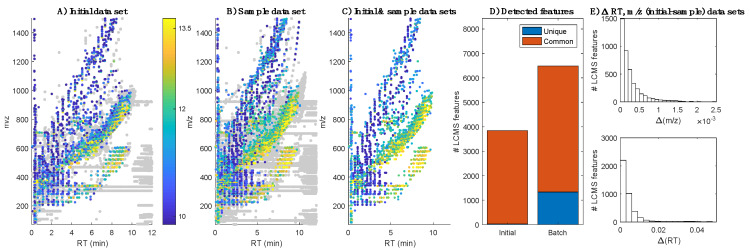
Distribution of background or noise features (grey dots) and informative (color dots, where the color represents the median log(intensity) in QCs) LC–MS features in the initial (**A**) and sample batches (**B**); (**C**) Distribution of features commonly detected in both data sets; (**D**) Number of unique and commonly detected features in the initial and sample batches; (**E**) Histograms of the differences in *m/z* (top) and RT (bottom) between features detected in both data sets.

Initial XCMS data pre-processing of data acquired from the ‘initial batch’ comprising the analysis of two blanks and three QCs described in the sample analysis in Section 3.4, identified 8971 LC–MS features. Among them, 4949 (56% of the total) were detected in blanks and classified as uninformative noise. Then, the ‘sample batch’—including 42 milk samples, 13 QCs (one QC every six samples) and three blanks—was analyzed. Peak table generation of data acquired from the analysis of the sample batch identified 18,401 features, of which 11,914 (65%) were classified as noise, leaving a total of 6487 features for further analysis. Figure 1 shows the distribution of detected and retained features (Figure 1A,B), the distribution of features commonly selected (Figure 1C) and the number of unique and commonly detected features (Figure 1D) in the initial and sample batches. The alignment of the retained LC–MS features in both subsets with 7.5 mDa and 0.1 min as *m/z* and RT tolerances, respectively, showed 4211 features that were commonly detected. Histograms depicted in Figure 1E also shows a very high similarity in both *m/z* and RT for those features commonly detected in both data sets, with 97% of the features showing differences in *m/z* and RT lower than 1 mDa and 0.05 min, respectively.

### 2.2. MS^2^ Data Dependent Acquisition Strategies

**Figure 2:**
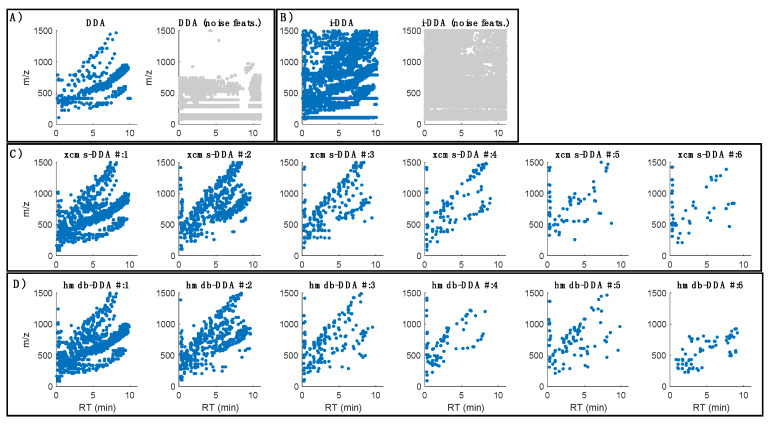
Distribution MS^2^ spectra acquired by DDA (**A**), i-DDA (**B**), xcms-DDA (**C**) and hmdb-DDA (**D**) assigned to informative (blue) and noise (grey) LC–MS features. Note: DDA: untargeted selection of precursors in the 70–1500 Da range; i-DDA: untargeted iterated DDA, in which MS^2^ spectra were acquired in seven QC replicates using DDA in the [70–200], [200–400], [40–600], [600–800], [800–1000], [1000–1250], and [1250–1500] Da ranges; xcms-DDA: targeted dynamic iterated DDA, in which precursor ions were selected using an inclusion list generated from the cleaned-up peak table obtained after the injection of two blanks and three QCs during system conditioning; hmdb-DDA: targeted dynamic iterated DDA using an inclusion list of (pre)annotated features in the cleaned-up peak table obtained after the injection of two blanks and three QCs during system conditioning.

Figure 2 shows the distribution of MS^2^ spectra acquired using the four considered strategies for data acquisition (DDA, i-DDA, xcms-DDA and hmdb-DDA) described in Section 3.5. MS^2^ Data Dependent Acquisition methods. A total of 3115 MS^2^ spectra were acquired using untargeted DDA in a single injection of a QC replicate. The use of seven QC injections in the i-DDA approach increased the number of acquired MS^2^ spectra up to 21,522. However, 16981 MS^2^ spectra (79% of the total) were assigned to noise or background features, a similar percentage to that observed in the case of DDA, where 2246 MS^2^ spectra (72% of the total) were assigned to uninformative features. After six QC injections, xcms-DDA lead to the acquisition of 3338 MS^2^ spectra (1736, 974, 331, 145, 92 and 60 MS^2^ spectra in each one of the consecutive LC runs) and hmdb-DDA allowed to the acquisition of 2993 MS^2^ spectra (1684, 777, 239, 99, 117 and 77 MS^2^ spectra, in each one of the consecutive LC runs). 

Targeted DDA reduced the number of precursors corresponding to uninformative features down to 31 and 30 in xcms-DDA and hmdb-DDA, respectively. In MS^2^ the precursor ions are sequentially selected from full scans and so, the number of spectra that can be acquired during a chromatogram is limited. Thus, the efficiency of MS^2^ acquisition depends not only on chromatographic (e.g., peak resolution, width, and symmetry) and sample parameters (e.g., distribution and intensity of features in the chromatogram), but also on instrumental parameters (e.g., scanning speed, MS^2^ acquisition time, sensitivity, isolation width), and on the strategy used for precursor selection. 

**Figure 3:**
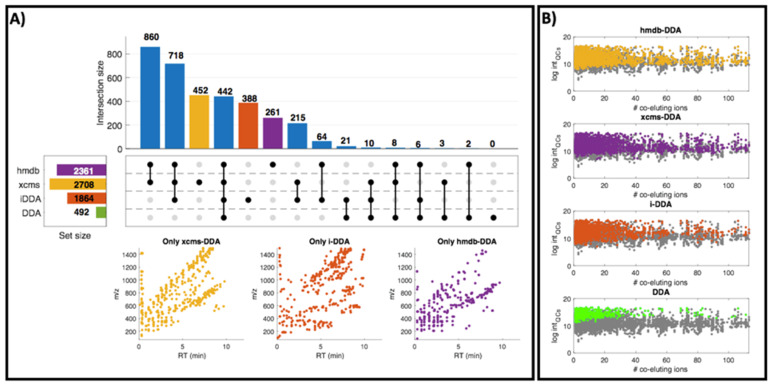
(**A-Top**) UpSet plot to visualize the intersecting sets of features selected as precursor for MS^2^ spectra acquisition using DDA, i-DDA, xcms-DDA or hmdb-DDA. (**A-Bottom**) Distribution of the 452, 388 and 261 features exclusively selected as precursors by xcms-DDA, i-DDA and hmdb-DDA, respectively. (**B**) Distribution of the median intensity values in the sample batch of the fragmented features selected using DDA, i-DDA, xcms-DDA and hmdb-DDA (color dots) and of features that were not fragmented (grey dots).

Figure 3A depicts results obtained from the analysis of the overlap among the fragmented features selected using DDA, i-DDA, xcms-DDA or hmdb-DDA using *m/z* and RT tolerances of 20 ppm and 0.1 min, respectively. Results showed an expected very poor performance of using a single LC-MS run with DDA and selection of precursors in the 70–1500 Da range. This method enabled the acquisition of MS^2^ spectra of 492 LC–MS features retained in the peak table obtained from the analysis of the milk samples and QCs (58 LC–MS runs, 6487 features), and no LC–MS feature was fragmented only by DDA. Results also showed a significant number of LC–MS features that were only fragmented using either xcms-DDA (452), hmdb-DDA (261) or i-DDA (388). 96% of the features fragmented by hmdb-DDA were also fragmented by xcms-DDA. 

Figure 3B displays the association between the number of co-eluting ions and the intensity of each feature in the sample batch. Data show that precursor selection in unsupervised DDA is biased towards the selection of high intensity ions with low number of co-eluting ions. This is a drawback because co-eluting, structurally similar compounds are frequently present in biological samples and one of the main advantages of using LC–MS based approaches is the ability of detecting low-abundant metabolites. Hence, the implementation of targeted DDA helped to circumvent this drawback notably.

**Figure 4:**
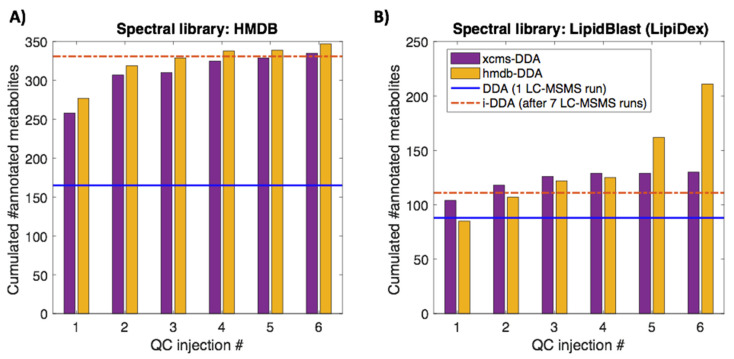
Number of annotated features in the sample batch using the HMDB (**A**) or LipidBlast (**B**) spectral libraries and MS^2^ spectra acquisition using DDA, i-DDA, xcms-DDA or hmdb-DDA.

Then, the impact of the precursor selection strategy pact on the metabolite annotation was evaluated. Figure 4 shows the number of acquired MS^2^ spectra and metabolites annotated as a function of the number of QC replicates used for data acquisition. Results indicate, in agreement with previous results, that even though DDA only requires the injection of one single QC sample, it is a highly inefficient approach that only enabled the annotation of 165 features (see Figure 4A). The set of 21522 MS^2^ spectra acquired by i-DDA was used for the identification of 331 metabolites after seven QC replicate injections. Results showed similar efficiencies of xcms-DDA and hmdb-DDA, in terms of the number of injections needed to reach a plateau in the number of annotated metabolites. In comparison to DDA and i-DDA, these approaches allowed to annotate a higher number of metabolites. In total, xcms-DDA and hmdb-DDA increased the number of annotated features up to 325 and 338, respectively, after four QC replicates, and 335 and 347, respectively, after six QC replicates. Similarly, using the LipidBlast library, i-DDA, xcms-DDA and hmdb-DDA outperformed DDA. The best results were obtained using hmdb-DDA and six QC injections, which allowed the annotation of 211 metabolites using the LipidBlast spectral library and a minimum spectral purity of 50, significantly larger than the 88, 111 and 130 annotations using DDA, i-DDA and xcms-DDA, respectively. Hmdb-DDA specifically targets for metabolites included in the database and so, it seems reasonable to find more annotated metabolites with more sampling. With xcms-DDA, on the other hand, more sampling does not guarantee a better coverage of HMDB metabolites but it may still improve coverage of the detected features to enable providing molecular structure identification of compounds not included in the database by using complementary tools, e.g., Sirius [5].

**Figure 5:**
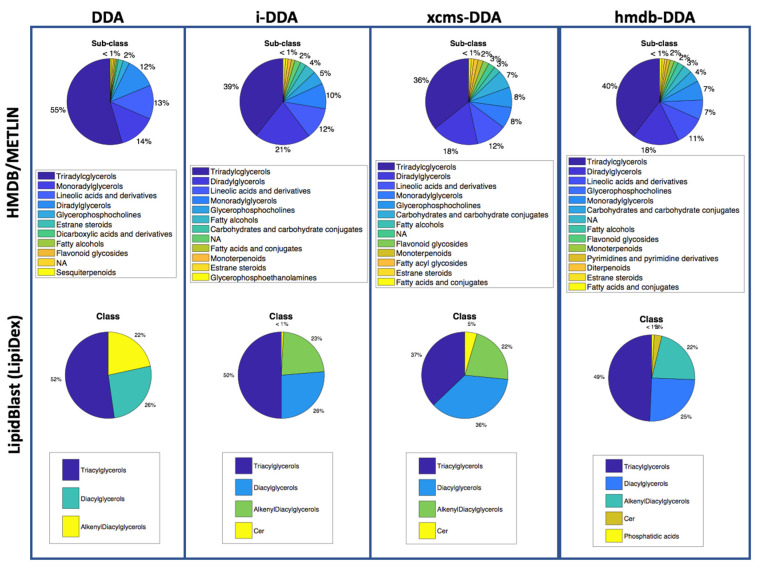
Distribution of the classes of metabolites annotated using the HMDB/METLIN or LipidBlast spectral libraries and spectra acquired by DDA, i-DDA, xcms-DDA or hmdb-DDA.

Figure 5 shows the distributions of classes of the annotated features using the HMDB/METLIN and LipidBlast spectral databases. Using the HMDB/METLIN as spectral library, the main classes of annotated metabolites were triacylglycerols, diacylglycerols, monoacylglycerols, glycerophosphocholines, lineolic acids and derivatives and fatty alcohols. Conversely, carbohydrates and carbohydrates conjugates, fatty acids and conjugates, and monoterpenoids were not annotated using untargeted DDA. Flavonoid glycosides were only annotated using targeted xcms-DDA or hmdb-DDA. Using LipidBlast, the main classes of detected metabolites were triacylglycerols, diacylglycerols and alkenyl-diacylglycerols. Ceramides were not annotated using DDA and phosphatidic acids were only annotated by hmdb-DDA.

### 2.3. MS^2^ Data Dependent Acquisition Strategies in QA/QC Pipelines

A number of projects and initiatives to establish minimum reporting guidelines and QC/QA procedures such as the ‘metabolomics standards initiative’ (Metabolomics Society), ‘COSMOS’ (FP7), MetExplore or PhenoMenal have been carried out in the last years to make metabolomic research more reproducible and generalizable. However, these guidelines are rarely adopted [6], contributing to the reproducibility crisis in science that affects metabolomics likely as much as any other area of research. Results presented here show that the use of straightforward iterative strategies—based on xcms-DDA or database guided-DDA (using HMDB as a model example), within standard QA/QC protocols—facilitates metabolite annotation by improving the MS^2^ coverage of informative features. Besides, these methods can be easily performed using standard open source software (e.g., R, matlab), facilitating the standardization of MS^2^ acquisition and, therefore, data comparison and reusability. Finally, from a practitioner’s perspective, a higher efficiency in the precursors selection will facilitate the adaptation of iterated DDA to include additional parameters to assess the quality of the acquired MS^2^ spectra. This way, instrumental parameters could be modified if required for a given LC–MS feature, to improve the spectral quality in subsequent LC–MS runs (e.g., by increasing the number of cumulated scans, modifying the collision energy set, or the MS isolation window).

## 3. Materials and Methods 

### 3.1. Standards and Reagents

LC–MS grade acetonitrile (ACN), isopropanol (IPA), methanol (MeOH), and methyl tert-butyl ether (MTBE) were obtained from Scharlau (Barcelona, Spain) and formic acid (≥95%), and ammonium acetate (≥98%) from Sigma-Aldrich Química SL (Madrid, Spain). Ultra-pure water was generated employing a Milli-Q Integral Water Purification System from Merck Millipore (Darmstadt, Germany). 

### 3.2. Research Ethics

All subjects gave their informed consent for inclusion before they participated in the study. The study was conducted in accordance with the Declaration of Helsinki, and the protocol was approved by the Ethics Committee for Biomedical Research of the Health Research Institute La Fe (Valencia, Spain) with approval number 2014/0247.

### 3.3. Sample Preparation

HM samples were provided by healthy volunteers admitted after the routine screening at the HM bank (*Banco de Leche Materna de la Generalitat Valenciana, Valencia, Spain*). Milk aliquots were collected directly before (N = 14) and after (N = 13) Holder pasteurization. In addition, HM samples from mothers of preterm infants (N = 15) were collected during their stay at the neonatal intensive care unit. HM samples were stored at -80 °C until their analysis. HM samples were thawed at room temperature followed by heating in a water bath at 33 °C for 10 min. Then, 5 μL of an internal standard (IS) solution containing oleic acid-D_9_ (80 µM) and prostaglandin F_2α_-D_4_ (39 µM) in H_2_O was added to 45 μL HM and then 175 µL MeOH followed by 175 µL MTBE was added to each sample [7]. The mixture was thoroughly shaken (1400 rpm) on a thermoblock mixer (20 °C, 1 min) and centrifuged at 4000 x *g* and 15 °C for 15 min. An amount of 20 µL of supernatant was added to 80 µL of a MeOH:MTBE (1:1, v/v) solution and then analyzed by UPLC–MS. A blank extract was prepared following the same procedure as described for HM samples, but replacing HM with water and a pooled QC sample was prepared by mixing 20 μL of each HM sample extract.

### 3.4. Sample Analysis

**Figure 6:**
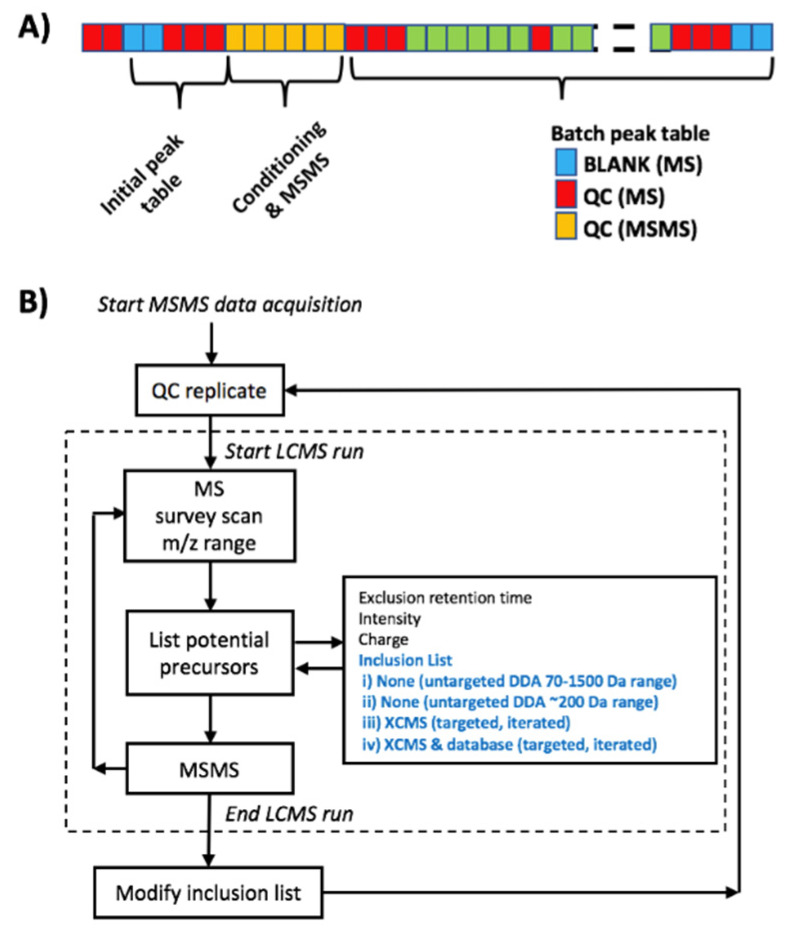
(**A**) Design of analytical batch integrating MS^2^ DDA approaches within an untargeted metabolomics workflow. (**B**) MS^2^ DDA approaches involving the use of QC replicates.

The experiment was designed to reproduce typical conditions for untargeted metabolomic experiments [1]. Accordingly, two blanks and a set of QCs were injected at the beginning of the sequence for system conditioning and MS^2^ data acquisition. Then, the sample batch including 42 milk samples, 13 QCs (1 QC every 6 samples) and 3 blanks were analyzed (Figure 6A). QCs were used to monitor the instrument performance, correct within-batch effects, and identify unreliable, background, and carry-over features as described elsewhere [8,9,10]. Untargeted metabolomic analysis was carried out employing a 1290 Infinity HPLC system from Agilent Technologies (CA, USA) equipped with a UPLC BEH C18 column (50 × 2.1 mm, 1.7 µm) from Waters (Wexford, Ireland). The flow rate was set to 400 µL min^−1^ running a binary mobile phase gradient starting at 98% of mobile phase A (5:1:4 IPA:MeOH:water, (5 mM ammonium acetate, 0.1% v/v formic acid)) for 0.5 min followed by a linear gradient from 2% to 20% of mobile phase B (99:1 IPA:water, (5 mM ammonium acetate, 0.1% v/v formic acid)) for 3.5 min and from 20% to 95% v/v of mobile phase B at 4 min; 95% v/v of mobile phase B was maintained for 1 min; the return to initial conditions was achieved after 0.25 min and was maintained for a total run time of 14 min. The column and autosampler were kept at 55 and 4 °C, respectively, and the injection volume was 2 µL.

### 3.5. MS^2^ Data Dependent Acquisition Methods

Two untargeted and two targeted DDA strategies for automated MS^2^ data acquisition based on the algorithm depicted in Figure 6B were employed: (i) untargeted selection of precursors in the 70–1500 Da range (DDA); (ii) untargeted iterated DDA, in which MS2 spectra were acquired in consecutive QC replicates using untargeted DDA in the [70–200], [200–400], [400–600], [600–800], [800–1000], [1000–1250], and [1250–1500] Da ranges (i-DDA); (iii) targeted dynamic iterated DDA, in which MS2 spectra were acquired by automated selection of precursor ions using an inclusion list generated after the injection of two blanks and three QCs during system conditioning (xcms-DDA). Here, LCMS features were classified as ‘informative’ and added to the inclusion list if the ratio between the minimum values in QCs and the maximum value in blanks was higher than 6; and (iv) targeted dynamic iterated DDA, where MS^2^ spectra were acquired using an inclusion list of (pre)annotated features after the injection of two blanks and three QCs during system conditioning (hmdb-DDA). In this case, LC–MS features were added to the inclusion list if they were not detected in blanks and could be (pre)annotated as a [M+H]^+^, [M+Na]^+^, [M+NH_4_]^+^, [M+H+Na]^+2^, [M+K]^+^, [M+H+K]^+2^, [M+H+CH_3_CN]^+^, [M+H+2CH_3_CN]^+^, [M+Na+CH_3_CN]^+^, [M+2Na-H]^+^, [2M+H]^+^, [2M+Na]^+^, [2M+K]^+^, [2M+NH_4_]^+^, [2M+H+CH_3_CN]^+^, [2M+Na+CH_3_CN]^+^, or [M+H-H_2_O]^+^ adduct of, at least, one of the 95688 metabolites included in the HMDB with a *m/z* accuracy error <20 ppm. Precursor and fragment ion tolerance should be selected depending on the mass accuracy of the MS, and wider ion tolerances (25–50 ppm) can be used for high abundant precursor ions [11]. In this study, 20 ppm *m/z* tolerances were selected in this study to limit false-positive peak detections.

Centroid mode at a rate of 5 Hz in the extended dynamic range mode (2 GHz), a collision energy set to 20 V, medium isolation window (~4 amu), MS^2^ fragmentation with automated selection of five precursor ions per cycle, and an exclusion window of 0.15 min after two consecutive selections of the same precursor were used in all cases.

For MS detection, an Agilent 6550 Spectrometer iFunnel quadrupole time-of-flight (QTOF) MS system working in the ESI^+^ mode was used. Full scan MS data in the range between 70 and 1500 *m/z* were acquired at a scan frequency of 5 Hz using the following parameters: gas T, 200 °C; drying gas, 14 L/min; nebulizer, 37 psi; sheath gas T, 350 °C; sheath gas flow, 11 L min^-1^. Mass reference standards were introduced into the source for automatic MS spectra recalibration during analysis via a reference sprayer valve using the 149.02332 (background contaminant), 121.050873 (purine), and 922.009798 (HP-0921) *m/z* as references.

### 3.6. Peak Table Generation and Metabolite Annotation

Peak table generation was carried out using XCMS software [12]. The *centWave* method was used for peak detection with the following parameters: mass accuracy, 20 ppm; peak width, (5,25); snthresh, 12; prefilter, (5,3000). A minimum difference in *m/z* of 7.5 mDa was selected for overlapping peaks. Intensity weighted *m/z* values of each feature were calculated using the *wMean* function. Peak limits used for integration were found through descent on the Mexican hat filtered data. Grouping before and after RT correction was carried out using the *nearest* method and 9 s as *rtCheck* argument. Finally, missing data points were filled by reintegrating the raw data files in the regions of the missing peaks using the *fillPeaks* method. The XCMS CAMERA [13] package was used for the identification of pseudospectra based on peak shape analysis, isotopic information and intensity correlation across samples. Each dataset was processed with CAMERA functions in the following order: *xsAnnotate*, *groupFWHM*, *findIsotopes*, *groupCorr* and *findAdducts* using standard parameters.

Identification and elimination of uninformative features was carried for each sample set independently. 

Metabolite annotation was carried out by matching experimentally acquired MS^2^ spectra with two MS^2^ databases: (i) a set of 241,952 experimental and predicted MS^2^ spectra of metabolites included in the HMDB without any pre-selection of MS resolution or collision energy and 25,653 MS^2^ spectra of 8945 metabolites from the METLIN database; and (ii) LipidBlast [11], a collection of in-silico ESI MS^2^ spectral libraries for the identification of neutral and polar lipid species developed from known theoretical fragmentations and experimental fragmentations and abundance information from MS^2^ spectra.

**Figure 7:**
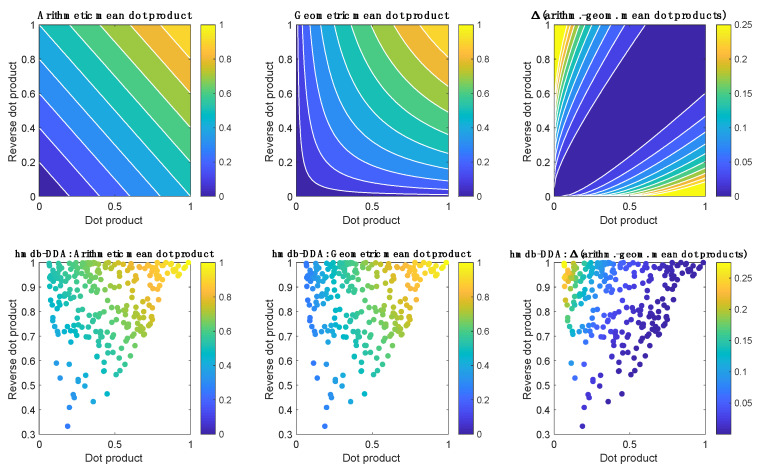
(Top) Surface plots of the arithmetic (left) and geometric (middle) means and the difference between the arithmetic and geometric means (right) of dot and reverse dot products in the 0–1 range. Bottom) Distribution of experimental *dp* and *rdp* values obtained in this study for LC–MS features annotated based on MS^2^ data acquired using hmdb-DDA. The color indicates the arithmetic (left) and geometric (middle) means, as well as the difference between the arithmetic and geometric means (right).

Two algorithms were used for annotation. Briefly, the first algorithm uses the HMDB/METLIN database first to determine for each fragmented feature whether the precursor ion can be (pre)annotated (*m/z* accuracy error<20 ppm) as the [M+H]^+^, [M+Na]^+^, [M+NH_4_]^+^, [M+H+Na]^+2^, [M+K]^+^, [M+H+K]^+2^, [M+H+ACN]^+^, [M+H+2ACN]^+^, [M+Na+ACN]^+^, [M+2Na-H]^+^, [2M+H]^+^, [2M+Na]^+^, [2M+K]^+^, [2M+NH_4_]^+^, [2M+H+ACN]^+^, [2M+Na+ACN]^+^ or [M+H-H_2_O]^+^ adduct of, at least, one metabolite included in the database. Then, the experimental MS^2^ spectrum is matched against the spectral database, after excluding ^13^C isotopologues. For each potential match, a spectral dot product (*dp*) and a reverse dot product (*rdp*) are calculated as similarity metrics representing the cosine of the angle between the experimental and reference spectral vectors as described elsewhere [14]. The calculation of the *rdp* only included ions present in both the experimental and reference spectra. Then, the geometric mean was calculated and the identity of the metabolites with the top *n* (n = 1 in this work) *mean dot products* are stored. The geometric mean provides slightly lower values than the arithmetic mean, except when both values are equal, in which case both means are equal. Figure 7 shows, as an example, surface plots of the arithmetic and geometric means (top) and the distribution of experimental *dp, rdp* and mean values (bottom) obtained using the complete set of 30,980 MS^2^ spectra acquired in this study. In situations when the *dp* is unusually low, the arithmetic mean can compensate a low score by a good score and so, the geometric mean can be used to reduce false positive annotations.

When an LC–MS feature is annotated, features included in the same pseudospectrum (i.e., CAMERA *pcgroup*), also detected in the experimental and reference MS^2^ spectrum (with *m/z* accuracy error<20 ppm, and an intensity above the absolute and/or relative threshold), are labelled as fragments of the annotated metabolite. Parameters for metabolite annotation include: *m/z* accuracy in both, precursor and fragment ions (20 ppm); the weight of *m/z* and intensity for the calculation of the *dp* and *rdp* [15] (in this study, m = 1.2 and n = 0.9 for *dp* and *rdp*, respectively, see Equation (1); the minimum number of matching ions in the experimental and reference spectra (in this study, 4) detected above user selected absolute and relative intensity thresholds (0.01% of the base peak and 500 AU, respectively), and a minimum *mean dp* (0.25, in this study). Furthermore, to reduce the effect of co-fragmented features in the score, the intensities of peaks present in the experimental but not in the reference MS^2^ spectrum were multiplied by 0.5 [16].

Equation (1): dot product between the reference and experimental spectra:

(1) $$dp=\frac{{\left(\sum^{}A_{exp}\cdot A_{ref}\right)}^{2}}{\sum^{}A_{exp}^{2}\cdot \sum^{}A_{ref}^{2}},$$

where A = (intensity)^m^(*m/z*)^n^.

Metabolite annotation using LipidBlast [11] was carried out using LipiDex as described elsewhere [15] using 0.01 Da tolerances in both MS (precursor) and MS^2^ (fragment) data and the ‘LipidBlast Acetate’ library.

### 3.7. Software and Data

Data acquisition and manual integration was carried out employing MassHunter Workstation (version B.07.00) from Agilent. Raw data (.D) was converted into mzXML format using ProteoWizard (http://proteowizard.sourceforge.net/). Peak detection, integration, deconvolution, alignment and pseudospectra identification, and inclusion lists generation were carried out using XCMS and CAMERA in R 3.6.1. Data analysis was carried out in MATLAB 2018b (Mathworks Inc., Natick, MA, USA) using in-house written scripts. Data and functions for annotation and data preprocessing scripts used in this work are available from the authors. Peak tables and MS^2^ data are also accessible via the Mendeley Data repository (https://data.mendeley.com/) under DOI:10.17632/fnzbxmkv83.1.

## 4. Conclusions 

In this work, we developed and compared targeted and untargeted DDA methods for metabolite annotation and compared results obtained in the frame of a QA/QC pipeline. The objective was to bring some light to the discussion concerning the selection of appropriate MS^2^ spectral acquisition methods providing a fast coverage at a low cost. Results obtained show that data acquired during the initial system conditioning enables a fast discrimination of relevant metabolic features and a more efficient selection of precursors. Furthermore, the iterative use of targeted DDA with inclusion lists of (pre)annotated metabolites further refines the list of precursor ions, reducing the number of LC runs required to achieve a given MS^2^ coverage of known metabolites. The improvement in the efficiency of the precursor efficiency provided by both types of targeted DDA facilitates their implementation within standard QA/QC pipelines, even during system conditioning. 

Publicly available data repositories such as the HMDB, METLIN or KEGG are constantly expanding and hence, the usefulness of the integration of such databases for automated DDA and peak annotation within untargeted metabolomics workflows may gain importance. However, an important limitation of using inclusion lists based on (pre)annotated metabolites for targeted DDA is that it excludes the acquisition of MS^2^ data of metabolites not included in the database at the time of the analysis. Thus, a routine application of targeted DDA methods such as xcms-DDA within standard metabolomic LC–MS apipelines, would enable the future re-analysis of data sets to improve and assess metabolite annotation, facilitating the reuse and joint analysis of multiple independent experiments, saving significant resources and leading to a more effective use of open science and collaborative work.

## References

[1] Broadhurst D., Goodacre R., Reinke S.N., Kuligowski J., Wilson I.D., Lewis M.R., Dunn W.B. (2018). Guidelines and considerations for the use of system suitability and quality control samples in mass spectrometry assays applied in untargeted clinical metabolomic studies. Metab. Off. J. Metab. Soc..

[2] Ivanisevic J., Want E.J. (2019). From samples to insights into metabolism: Uncovering biologically relevant information in LC-HRMS metabolomics data. Metabolites.

[3] Mullard G., Allwood J.W., Weber R., Brown M., Begley P., Hollywood K.A., Jones M., Unwin R.D., Bishop P.N., Cooper G.J.S. (2015). A new strategy for MS/MS data acquisition applying multiple data dependent experiments on Orbitrap mass spectrometers in non-targeted metabolomic applications. Metabolomics.

[4] Wang Y., Feng R., He C., Su H., Ma H., Wan J.-B. (2018). An integrated strategy to improve data acquisition and metabolite identification by time-staggered ion lists in UHPLC/Q-TOF MS-based metabolomics. J. Pharm. Biomed. Anal..

[5] Dührkop K., Fleischauer M., Ludwig M., Aksenov A.A., Melnik A.V., Meusel M., Dorrestein P.C., Rousu J., Böcker S. (2019). Sirius 4: A rapid tool for turning tandem mass spectra into metabolite structure information. Nat. Methods.

[6] Considine E.C., Thomas G., Boulesteix A.L., Khashan A.S., Kenny L.C. (2017). Critical review of reporting of the data analysis step in metabolomics. Metabolomics.

[7] Villaseñor A., Garcia-Perez I., Garcia A., Posma J.M., Fernández-López M., Nicholas A.J., Modi N., Holmes E., Barbas C. (2014). Breast milk metabolome characterization in a single-phase extraction, multiplatform analytical approach. Anal. Chem..

[8] Martínez-Sena T., Luongo G., Sanjuan-Herráez D., Castell J.V., Vento M., Quintás G., Kuligowski J. (2019). Monitoring of system conditioning after blank injections in untargeted UPLC-MS metabolomic analysis. Sci. Rep..

[9] Kuligowski J., Sánchez-Illana Á., Sanjuán-Herráez D., Vento M., Quintás G. (2015). Intra-batch effect correction in liquid chromatography-mass spectrometry using quality control samples and support vector regression (QC-SVRC). Analyst.

[10] Sánchez-Illana Á., Pérez-Guaita D., Cuesta-García D., Sanjuan-Herráez J.D., Vento M., Ruiz-Cerdá J.L., Quintás G., Kuligowski J. (2018). Model selection for within-batch effect correction in UPLC-MS metabolomics using quality control—Support vector regression. Anal. Chim. Acta.

[11] Kind T., Liu K.-H., Lee D.Y., DeFelice B., Meissen J.K., Fiehn O. (2013). LipidBlast in silico tandem mass spectrometry database for lipid identification. Nat. Methods.

[12] Smith C.A., Want E.J., O’Maille G., Abagyan R., Siuzdak G. (2006). XCMS: Processing mass spectrometry data for metabolite profiling using nonlinear peak alignment, matching, and identification. Anal. Chem..

[13] Kuhl C., Tautenhahn R., Böttcher C., Larson T.R., Neumann S. (2012). Camera: An integrated strategy for compound spectra extraction and annotation of liquid chromatography/mass spectrometry data sets. Anal. Chem..

[14] Stein S.E., Scott D.R. (1994). Optimization and testing of mass spectral library search algorithms for compound identification. J. Am. Soc. Mass Spectrom..

[15] Hutchins P.D., Russell J.D., Coon J.J. (2018). LipiDex: An Integrated Software Package for High-Confidence Lipid Identification. Cell Syst..

[16] Tsugawa H., Cajka T., Kind T., Ma Y., Higgins B., Ikeda K., Kanazawa M., VanderGheynst J., Fiehn O., Arita M. (2015). MS-DIAL: Data-independent MS/MS deconvolution for comprehensive metabolome analysis. Nat. Methods.

